# Exploring healthcare providers’ perspectives on virtual care delivery: insights into telemedicine services

**DOI:** 10.1186/s12913-023-10244-w

**Published:** 2024-01-02

**Authors:** Israa K. Abdelghany, Ranim AlMatar, Asmaa Al-Haqan, Israa Abdullah, Salah Waheedi

**Affiliations:** Department of Pharmacy Practice, College of Pharmacy, Kuwait University, Jabriya, Kuwait

**Keywords:** Telemedicine, Health care providers, Questionnaire, Interviews, COVID-19

## Abstract

**Background:**

The rapid advancement of technology has led to a concurrent advancement of telemedicine, that is the delivery of medical services over a long distance using technological methods. The consistently growing numbers of COVID-19 cases warranted the use of telemedicine as an alternative method of care-delivery. This study aims to evaluate perceptions of healthcare services provided virtually among healthcare providers (HCPs) in Kuwait and to assess their acceptance and intention to implement such services.

**Methods:**

An exploratory mixed methods design was conducted, where in phase one HCPs’ perceptions towards telemedicine were explored through an online questionnaire and the quantitative data were summarized by descriptive analysis using SPSS. Scores for usefulness and for attitude toward the use of telemedicine were calculated. Then semi-structured interviews were conducted in phase two and the qualitative data were analyzed thematically.

**Results:**

In phase one, 421 HCPs answered the questionnaire***.*** In terms of telemedicine knowledge, 15.4% of HCPs had previously used telemedicine technology and 39% already knew about it. Additionally, 42.3% preferred to use telemedicine, and 88.5% had a moderate to high usefulness score. Telemedicine’s ease of use was perceived positively. Attitude median score was 73 with an IQR of 16 (63–79). Half of the participants intend to use telemedicine.

In phase two, twenty-two interviews were conducted resulting in six themes; HCPs’ acceptance of telemedicine, facilitators and motives for telemedicine implementation, skills and training required to conduct telemedicine, barriers limiting the use of telemedicine, strategies to overcome the barriers, and benefits of telemedicine.

**Conclusion:**

Most of the HCPs favor telemedicine integration into Kuwait’s healthcare system as their perceptions of telemedicine were overall positive, paving the way to develop implementation strategies.

**Supplementary Information:**

The online version contains supplementary material available at 10.1186/s12913-023-10244-w.

## Background

Governments and health organizations worldwide have been developing services and regulations dedicated to providing the general population with a high-quality healthcare system. This system aims to reduce the burden of disease or injury, disability, and to improve peoples’ overall health status, as well as functioning level. However, booming public expectations, transforming health-related needs, and new aspiring goals are raising the bar for health systems to aim for better health outcomes. For that to be achieved, a high-quality health system is needed to optimize healthcare by the continuous provision of care, thus, improving or maintaining health [1]. One of the ways healthcare can be improved is by implementing different forms of technology [2]. Since we are living in a digital world, where communications, computer sciences, informatics, and medical technologies continue to progress rapidly, one must have heard about the term telehealth or telemedicine. As the name implies, the prefix tele- from Greek *telos*- means at a distance and health or medicine refers to different kinds of services and outcomes related to healthcare [3]. Throughout the past few decades, the swift development in technology led to a concurrent telemedicine advancement, resulting in a sophisticated targeted service used in homes, hospitals, and other healthcare facilities [2].

According to the World Health Organization (WHO), telemedicine is defined as “the provision of healthcare services at a distance with communication conducted between healthcare providers seeking clinical guidance and support from other healthcare providers (provider-to-provider telemedicine) or conducted between remote healthcare users seeking health services and healthcare providers (client-to-provider telemedicine)’’ [3]. Televisits are healthcare services provided through telemedicine and are defined as medical acts in which doctors communicate with patients remotely and might result in medicine or treatment prescriptions [3, 4]. In our study, we will use the term ‘telemedicine’ and choose ‘televisits’ as the healthcare service under question.

The rapidly spreading Coronavirus Disease 2019 (COVID-19) led to devastating consequences on healthcare systems, health workers, and patients, as well as the economy worldwide [5]. The challenge of the consistently growing number of COVID-19 cases and deaths warranted the establishment of an alternative method of care delivery and disease control. The emergence of COVID-19 was a turning point in the journey of telemedicine into practice [6]. During the pandemic, social isolation and extreme lockdown measures were implemented, this resulted in using telemedicine not only to take care of COVID-19 patients, but also in public awareness, research, and teaching [7]. These versatile areas of applications suggest using telemedicine even after the COVID-19 pandemic is over, due to the enhanced HCPs’ perception of such technology [8].

Telemedicine is considered a viable option by patients and healthcare professionals for providing healthcare services. Quality of care and confidentiality, as well as ensured privacy, are prerequisites for patients to accept telemedicine [9]. Moreover, telemedicine use would save time and is cheaper for patients considering the transportation cost, unpaid leaves, and gas prices [4]. However, the presence of perceived barriers to telemedicine, for example, the lack of physical examination and problems with the devices used, is linked to low utilization.Moreover, HCPs’ perceptions of this care delivery method are also a crucial factor in adoption. The importance of telemedicine in Kuwait, however, has not yet been properly established.

This study aims to evaluate the perceptions of HCPs for healthcare services provided virtually in Kuwait, and to assess their acceptance and intention to implement this service.

## Methods

### Study design and setting

This study was conducted using an exploratory sequential mixed methods design [10, 11]. The healthcare providers’ perception towards telemedicine was explored using a quantitative online questionnaire (Phase 1). The findings from the questionnaire were further explored by conducting semi-structured interviews with participants who choose to provide their contact details for phase 2. Finally, findings from both phases were interpreted through integration of the quantitative and qualitative data.

This study was carried out in Kuwait, which has a population of around 4.1 million. The healthcare system in Kuwait provides primary, secondary, and tertiary healthcare services in both public (government) and private sectors. During COVID-19 healthcare facilities in the governmental and private sector started to provide some services virtually. However, some of these services stopped shortly after health restrictions were removed.

### Sample size and sampling strategy

For the online questionnaire (Phase 1), sample size was calculated using the Raosoft sample size calculator [12] with chosen 5% as a margin of error, 95% as a confidence interval (CI), and a response distribution of 50%. According to the Central Statistical Bureau in Kuwait (CSB), the 2020 statistical analysis of the Ministry of Health (MOH) workers showed that there are 61,993 healthcare providers (CSB, 2020). “Raosoft.com” stated a minimum sample of 382 is required to have statistically meaningful results. A chain-referral convenient sampling method was used to recruit healthcare providers.

For the semi-structured interviews (Phase 2), the participants were recruited through the survey, as well as through snowball sampling. To obtain a range of perspectives, the interviews included healthcare professionals across different roles in the healthcare system, including doctors, pharmacists, nurses, and allied health professionals from several practice sites. Participants could either be working in the public or private sector, or self-employed. Participants were excluded if they were not actively providing any healthcare service or if they were working outside of Kuwait at the time of the study.

### Phase 1: online survey

An online survey study was conducted using a self-administered questionnaire. Participants were provided with information on the purpose of the study on the first page of the questionnaire. Prior to commencing the online survey, several measures were taken to ensure the confidentiality of the participants’ data. These included collecting responses anonymously through a secure platform, refraining from requesting any personal identifiable information, and obtaining informed consent from all participants. The participants were required to indicate their willingness to engage in the study by selecting either the option 'I agree to participate' or 'I do not agree to participate'. Participants who opted for the latter choice were promptly redirected to the conclusion of the questionnaire, thus excluding them from further participation.

#### Questionnaire development

The questionnaire was adopted from Horne et. al, following a literature review [13]. The questionnaire was composed of six sections. Section 1: participants’ demographics, Section 2: perceived usefulness (7 statements), Section 3: perceived ease of use (6 statements). Section 4: providers’ attitude toward telemedicine (21 statements), and Section 5: intention to use telemedicine (6 statements). Sections 2 to 5 included Likert scales ranging from 1 = strongly disagree to 5 = strongly agree. The questionnaire ended with an optional question requesting participants' phone number for participation in a semi-structured interview to further investigate their perceptions of telemedicine.

The survey was designed and distributed through Qualtrics Survey Software (QSS) (Qualtrics, Provo, UT, USA). Before distribution, the questionnaire was translated into Arabic language and piloted on 10 participants (3 doctors, 2 nurses, 5 pharmacists) and minor edits were done, mainly to the general format and navigation through questions. Furthermore, some vocabularies were changed to be easily understood.

### Phase 2: semi-structured interviews

HCPs who participated in phase 1 and provided their contact details were invited to participate in phase 2, a semi-structured interview. The interview aimed to obtain an in-depth understanding of the HCPs' perspectives on telemedicine in Kuwait, patterns of its adoption, and use in the Kuwaiti healthcare system. The interviews were conducted by the first author.

#### Semi-structured interviews guide and setting

The interviews were based on a predetermined interview guide that was designed to elicit healthcare professionals’ perceptions regarding the utility of, and barriers to, delivery of telemedicine services in Kuwait. The initial interview guide was drafted by R.A. and I.A. based on previous literature and was reviewed by the research team [7, 14]. The guide was piloted with a pharmacist for accuracy, and the pilot interview was included in the results.

Interviews were conducted via telephone calls and took place between 21^st^ of April 2022 to the 9^th^ of May 2022. The interviews lasted for an average of 20 min. The concept of theoretical saturation was followed. After twenty-two interviews, no new ideas emerged, and saturation was judged to be achieved by the investigators.

#### Data collection and analysis

For phase 1, an anonymous questionnaire link was distributed online using social media platforms, including WhatsApp, Twitter, Instagram, and Microsoft Teams between April and May 2021. Reminders were sent weekly using the same platforms, and the questionnaire was closed after reaching the target sample size. Regarding phase 2, the semi-structured interviews, participants were provided with information on the purpose of the study, and verbal consent for participation and recording was granted prior to starting the interviews. All interviews were recorded for accuracy and transcribed verbatim.

Quantitative data were analyzed using Statistical Package for the Social Sciences (SPSS®) version 27.0, Armonk, NY: IBM Corp. Normality of data was investigated using Kolmogorov–Smirnov, which warranted the use of non-parametric tests. Demographic data is presented as frequencies, percentages, median, and interquartile range (IQR). For sections 2–5, scores for perceived usefulness, perceived ease of use, provider’s attitude toward telemedicine, and intention to use telemedicine were calculated for each participant based on the Likert Scale (1 = strongly disagree, 2 = disagree, 3 = neutral, 4 = agree, 5 = strongly agree). Some statements were negatively worded, so scores were reversed, giving 5 points to strongly disagree, going down to 1 point given to strongly agree. For easier navigation through tables, we combined 1 and 2, as well as 3 and 4 in Likert sales. According to their scores, participants were divided into three groups: low (8-18), moderate (19-29), and high (30–40). Spearman's test was used to test correlation between dependent and independent variables, followed by cross-tabulation. Statistical significance was accepted with a *P*-value < 0.05.

A thematic analysis method, including both deductive and inductive approaches, was used to organize, interpret, and generate themes from qualitative data; which facilitated the discovery of the perceptions of each HCP by analyzing their responses [15]. This study used the qualitative data analysis software MAXQDA 22.2.0 to store, classify, and code all transcripts. The process of thematic analysis started with data familiarization, which involved reading and re-reading the data as well as re-listening to parts of the audio recordings. The data were coded with initial codes ranging from words to sentences. After the preliminary coding, the similarities and differences between these codes were compared, assigning the codes to potential themes, which were the initial themes. The refinement of these themes involved re-organizing the existing ones, generating new themes, and removing those that lacked relevance. At each stage of refinement, the I.K.A. and I.A. reviewed and discussed the potential themes until a final definition and naming of each theme and sub-theme were agreed upon.

#### Ethical considerations

This study was approved by the Joint Health Science Centre and Ministry of Health Research Ethical Committee, Kuwait, (MoH/REC/1385) and (MoH/REC/196/2022). All methods were performed in accordance with the relevant guidelines and regulations. Informed consent was obtained from all subjects and/or their legal guardian(s) for this study.

## Results

### Phase 1

A total of 657 participants accessed the survey link, however, only 421 answered the questionnaire. The majority of participants (*N* = 255, 60.6%) were aged between 22 and 40 years old, with a median age of 35 years and an interquartile range (IQR) of 20 (28–47.5). The number of female participants was 280 (66.5%). The nationality of participants was almost equally divided into Kuwaitis (*N* = 206, 48.9%) and Non-Kuwaitis (*N* = 212, 50.4%). Bachelor's degree was the highest obtained level of education comprising 129 (45.6%) participants. The majority of participants were either physicians (*N* = 195, 46.3%) or pharmacists (*N* = 141, 33.5%). Participants' demographic characteristics are presented in Table 1.

**Table 1:** Sociodemographic characteristics and occupational information

**Demographics:**	***N***	**%**
***Age groups:***		
** 22–30**	139	33
** 31–40**	116	27.6
** 41–50**	83	19.7
** 51–69**	83	19.7
***Gender:***		
** Female**	280	66.5
** Male**	139	33
***Highest degree obtained:***		
** Diploma**	47	11.2
** Bachelor's degree**	129	45.6
** Master's degree**	107	25.4
** Ph.D**	52	12.4
** Other**	19	4.5
***Communication skills in Arabic:***		
** Good/Very Good**	399	94.8
** Neither Good nor Poor**	16	3.8
** Poor**	3	0.7
***Communication skills in English:***		
** Good/ Very Good**	394	93.6
** Neither Good nor Poor**	19	4.5
** Poor/ Very poor**	5	12
***Profession:***		
** Physician**	195	46.3
** Pharmacist**	141	33.5
** Nurse**	35	8.3
** Nutritionist**	5	1.2
** Lab technician**	6	1.4
** Other:**	37	8.8
**Occupational information:**		
***Working health area:***		
**Hawally**	130	30.9
**AL-Asimah (Capital)**	79	18.8
**Ahmadi**	36	8.6
**Farwaniya**	73	17.3
**Jahra**	13	3.1
**Mubarak AL-Kabeer**	20	4.8
**Al-Sabah**	59	14
***Practice site:***		
**Governmental hospital**	343	81.5
**Private hospital**	32	7.6
**Other:**	42	10

In regard to participants’ familiarity with telemedicine, a minority of respondents (*N* = 55, 13.1%) reported never having heard about it, while 65 HCPs (15.4%) had prior experience using telemedicine technology. Of all participating HCPs, 178 (42.3%) prefer to use telemedicine, whereas 60 (14.3%) participants prefer not to use it.

The assessment of perceived usefulness resulted in a median score of 26 and an IQR of 6 (22-28). Three hundred and twenty-three respondents (88.5%) had a moderate to high usefulness score, scored by 172 (46.5%) and 154 (42%) participants respectively. On the other hand, only 42 (10%) participants had a low usefulness score. More than half of HCPs thought using telemedicine would enable them to accomplish tasks quickly (*N* = 238, 56.6%) and help in multiple aspects of patient care (*N* = 250, 59.4%).

Regarding telemedicine's perceived ease of use, perceptions were overall positive, with almost half of respondents agreeing or strongly agreeing that it is facile to operate, control, and interact through telemedicine. Results showed that 17 (4.6%) participants had a low ease-of-use score, while 170 (46.1%) had a moderate score and 181 (49.2%) had a high score. This section had a median score of 22 and an IQR of 4 (20-24). Most participants believed it is easy to operate (*N* = 280, 66.5%) telemedicine and become skillful (*N* = 240, 57%) using this technology.

Six statements exploring HCPs' attitudes toward telemedicine resulted in a median score of 73 and an IQR of 16 (63–79). More than half of the respondents (*N* = 214, 62.5%) had a moderate attitude score and 36.3% (*N* = 124) had a high score, whereas only 4 (1.2%) participants scored low.

By probing HCPs' intention to use telemedicine, we found only 13 (3.9%) participants had a low use-intention score, while the remaining scored between moderate (*N* = 147, 44.9%) and high (*N* = 167, 51.1%). The providers' use-intention median score was 23 with an IQR of 4 (20-24). Further details are provided in Table 2.

**Table 2:** HCPs' Perception of Telemedicine

	Scale		
Statements	^a^¨Disagree Strongly disagree	Uncertain	^b^Agree Strongly agree
*Telemedicine perceived ability to enhance patient care:*			
**Using telemedicine would enable me to accomplish tasks more quickly**	41 (9.7%)	90 (21.4%)	238 (56.6%)
**Using telemedicine would improve my job performance**	63 (15%)	125 (29.7%)	180 (42.8%)
**Using telemedicine in my job would increase my productivity**	52 (12.3%)	109 (25.9%)	207 (49.2%)
**Using telemedicine would enhance my effectiveness on the job**	60 (14.2%)	106 (25.2%)	202 (47.9%)
**Using telemedicine would make it easier to do my job**	47 (11.3%)	90 (21.4%)	231 (54.8%)
**I would find telemedicine useful in my job**	50 (11.9%)	93 (22.1%)	225 (53.4%)
**Using telemedicine would improve communication on my job**	51 (12.1%)	94 (22.3%)	223 (53%)
*Perceived telemedicine ease of use:*			
**Learning to operate telemedicine technology would be easy for me**	17 (4%)	72 (17.1%)	280 (66.5%)
**I would find it easy to get telemedicine technology to do what I want it to do**	23 (5.5%)	114 (27.1%)	231 (54.9%)
**My interaction with telemedicine technology would be clear and understandable**	24 (5.7%)	124 (29.5%)	220 (52.3%)
**I would find telemedicine technology to be flexible to interact with**	36 (8.5%)	126 (29.9%)	206 (48.9%)
**It would be easy for me to become skillful at using telemedicine technology**	26 (6.2%)	102 (24.2%)	240 (57%)
**I would find telemedicine technology easy to use**	26 (6.2%)	119 (28.3%)	223 (52.9%)
*Attitude towards telemedicine*			
**The use of telemedicine improves patient care by giving the provider more time with the patients**	46 (10.9%)	81 (19.2%)	216 (51.3%)
**Telemedicine can be adapted to assist providers in many aspects of patient care**	26 (6.2%)	66 (15.7%)	250 (59.4%)
**A telemedicine system offers providers a remarkable opportunity to improve patient care**	38 (9%)	101 (24%)	203 (48.3%)
**Telemedicine technology represents a violation of patient privacy**	192 (45.6%)	99 (23.5%)	51 (12.1%)
**Telemedicine technology causes providers to give less time to quality patient care**	106 (25.2%)	105 (24.9%)	131 (31.2%)
**Telemedicine increases cost by increasing the provider’s workload**	121 (28.8%)	150 (35.6%)	71 (16.9%)
**It takes as much effort to maintain patient records using telemedicine technology as it does by hand**	149 (35.4%)	82 (19.5%)	111 (26.3%)
**Telemedicine creates more problems than they solve in providing health care**	142 (33.8%)	125 (29.7%)	75 (17.8%)
**The use of telemedicine dehumanizes patient care**	136 (32.3%)	113 (26.8%)	93 (22.1%)
**Part of the increase in costs of health care is because of telemedicine**	126 (29.9%)	164 (39%)	52 (12.1%)
**Confidentiality will not be sacrificed by using telemedicine**	51 (12.1%)	93 (22.1%)	198 (47.1%)
**I would be comfortable using telemedicine**	38 (9%)	103 (24.5%)	201 (47.7%)
**Working with telemedicine technology would make me very nervous**	172 (40.9%)	107 (25.4%)	63 (15%)
**I feel threatened when others talk about telemedicine**	209 (49.7%)	85 (20.2%)	48 (11.4%)
**Telemedicine technology does not scare me at all**	22 (5.3%)	74 (17.6%)	246 (58.5%)
**I feel hostile toward using telemedicine**	225 (53.5%)	67 (15.9%)	50 (11.9%)
**Telemedicine makes me feel uneasy and confused**	230 (54.6%)	63 (15%)	49 (11.7%)
**I have a lot of self-confidence when it comes to working with telemedicine**	38 (9%)	114 (27.1%)	190 (45.1%)
**Confidentiality is nearly impossible if patient records are used during telemedicine**	186 (44.2%)	104 (24.7%)	52 (12.4%)
**Providing health care does not lead itself to using telemedicine**	72 (17.1%)	198 (47%)	72 (17.1%)
**Telemedicine would make providers’ jobs easier**	27 (6.5%)	91 (21.6%)	224 (53.2%)
*Intention to use telemedicine to provide patient care:*			
**I intend to use telemedicine with my patient care and management when it is available in my clinic or hospital**	26 (6.2%)	54 (12.8%)	247 (58.7%)
**I intend to use telemedicine technology to provide healthcare services to patients as often as needed**	17 (4%)	46 (10.9%)	264 (62.7%)
**I intend NOT to use telemedicine in my patient care and management routinely**	169 (40.1%)	74 (17.6%)	84 (20%)
**Whenever possible, I intend NOT to use telemedicine in patient care and management**	203 (48.3%)	78 (18.5%)	46 (11%)
**To the extent possible, I would use telemedicine technology to do different things, clinical or non-clinical**	23 (5.5%)	75 (17.8%)	229 (54.4%)
**To the extent possible, I would use telemedicine in my patient care and management frequently**	20 (4.8%)	65 (15.4%)	242 (72.2%)

^a^"Disagree" and "strongly disagree" are merged

^b^"Agree" and "Strongly agree" are merged

The effect of attitude towards telemedicine, perceived ease of use, and usefulness on use intention were investigated using Spearman's test. Results showed significant correlations, especially between attitude towards telemedicine and use-intention (*r* = 0.495, *p* < 0.001). Another factor that influenced use intention and preference was knowledge of telemedicine or previous experience. Further details are provided in Table 3.

**Table 3:** Significant correlations:

Factors:	*P*	r
Attitude *and* Use intention	< 0.001	0.495
Usefulness *and* Use intention	< 0.001	0.439
Ease of use *and* Use intention	< 0.001	0.341
Knowledge or experience *and* Use preference	< 0.001	0.28
Knowledge or experience *and* Use intention	< 0.001	0.117

The significance was confirmed if *p *<0.05

### Phase 2

Twenty-two interviews involving nine physicians, eight pharmacists, three allied health professionals, and two nurses were conducted. We identified 6 themes expressed by the healthcare providers. These themes were (1) the overall acceptance of telemedicine; (2) Facilitators and motives for telemedicine implementation; (3) Skills and training required to conduct televisits; (4) Barriers limiting the use of telemedicine; (5) Strategies to overcome the barriers; and (6) Benefits of telemedicine.

#### Theme 1: the overall acceptance of telemedicine

Telemedicine was accepted by HCPs because it was perceived as an alternative method to face-to-face healthcare services. As stated by one participant, *“I was hoping for the service to resume later on because it will save too much and save time on the physicians, nurses, and pharmacists in the polyclinics” (P1, pharmacist).* A physician mentioned that the doctor’s specialty may determine their acceptance of telemedicine use in their practice. Another physician said, *“But for me as an ENT surgeon… working in ENT for about 14 years. I believe if I were still there and have these e-services, I would never rely on them 100% because I need to see the patient” (P5, physician).* Although some HCPs accept televisits, some providers are resistant to change because of the increased workload. Moreover, in our interviews, we assessed the acceptance of video calls for various purposes, such as conducting patient visits, demonstrating a product or a tool, or counseling patients. While video calls can facilitate many aspects of healthcare, in some areas, a complete subjective and objective assessment may not be feasible through video calls. Some interviewees were supportive of its use, while others were not, and some found that it depends on patients’ preferences. A physician reported, *“for example, in the beginning, they don’t show us how they look, and then they open the camera the next day” (P2, physician).*

#### Theme 2: facilitators and motives for telemedicine implementation

The interviews revealed that the providers in Kuwait frequently used WhatsApp, Zoom, Microsoft Teams, and regular phone calls or text messages. It is important to use platforms compatible with the Health Insurance Portability and Accountability Act (HIPAA) requirements. This avoids patients’ privacy and confidentiality concerns. Lastly, the availability of good internet connectivity, a high-quality device, a private room, and administrative support made it easier to perform televisits. As mentioned by participant 9, *“[Important thing] to have good internet service and we have also connected many hospitals with our system to see x-rays by the civil ID of the patient this also good it is like communicating online with other hospitals and patients” (P9, physician).* Regarding the motives, patients’ sake was the one mostly mentioned. As one of the participants said, *“For patient’s sake- the reason that made me start telemedicine back then was a patient who was working in a bank and cannot come for visits in the morning and if he lost follow-up his health may deteriorate, that’s when we thought of telemedicine… that telemedicine or e-Health then will be a good option for them” (P2, physician).* In addition, participants 15, 16, and 22 stated that being trained before has encouraged them to incorporate telemedicine in their practice.

#### Theme 3: skills and training required to conduct televisits

Telemedicine relies on training and the development of specific skills to eliminate errors and increase the quality of care. Many healthcare professionals mentioned that it is essential for an HCP to have good communication skills, active listening, ethics, and good training to be qualified to use telemedicine. In our interviews, we only asked the participants who had already tried telemedicine before if they thought that training was required, and 8 out of 11 said yes. However, those who opposed training emphasized the ease of which platforms such as Zoom and WhatsApp may be used. In that regard, a participant reported, *“For example, there is a program that is public… such as Zoom or Teams, everybody can learn about them easily through YouTube or searching the internet, but some programs are related to the ministry of health, and it is developed by the ministry of health, and these require training for the healthcare providers before using it.” (P17, pharmacist).*

#### Theme 4: barriers limiting the use of telemedicine

The participating healthcare professionals had many perspectives on barriers, which were influenced by three factors: Clinical or provider, technological, and patient factors. The clinical or provider factors can be related to the institution itself or the HCPs. Regardless of the HCPs’ profession, they all had issues examining patients, also mentioned as lack of physical examination, because it led to underestimating the diagnosis. In our interviews, an HCP said, *“In acute conditions, it cannot be applied because it needs physical examination, and there was a teleconsultation between the doctor and patient during corona, but the doctor was not able to diagnose or measure the vitals of the patients so many of the patients got deteriorated” (P11, pharmacist).* One participant questioned the ethics of this practice, claiming that the quality of care offered is inferior to in-person visits. Regarding this finding, the interviewee said, *“This is not ethical because the quality will not be as good as when I see the patient and give him/her my time and physically examine him/her” (P9, physician).* This means that the equity of care will not be achieved. In Kuwait, there is a shortage in staff, which may lead to an increase in workload for healthcare professionals. Moreover, currently there is no ministerial rule governing this service’s implementation.

Technological factors are the second category considered as barriers to implementing televisits. Problems with the device or program utilized and the internet connection are some of these technical issues that influence telemedicine acceptance. In practice here in Kuwait, problems regarding the internet connection and having a device specified for the service were stated. An HCP said, *“we have many people from the clinical staff that are illiterate about technology” (P2, physician)* which necessitates training, as mentioned in theme 2. Furthermore, there are no chief technology officers to assist HCPs in some institutions.

The final factor limiting telemedicine acceptance is patient-related issues. According to the providers, patients’ preferences were mentioned as a barrier because many prefer in-person appointments to online televisits. This was mainly addressed in elderly patients and could be attributed to mistrust or a lack of conviction in telemedicine. Several participants mentioned this and one of them added, *“We have barriers or difficulties with the patients, some elderly don’t like to use the technology and even some patients who are not elderly, but they don’t prefer to use technology, so it is about patient preference and patients’ knowledge to use the technology’’ (P17, pharmacist).* Moreover, one HCP stated that the reason behind geriatrics and pediatrics being the most challenging age groups to assess during televisits is that they have difficulties in communication. In geriatrics, this could be due to hearing and visual problems. Additionally, some patients refuse to appear on camera because they fear being recorded. Finally, both HCPs and patients should have strong communication skills, as noted in theme 2, to be able to communicate with each other well.

#### Theme 5: strategies to overcome the barriers

Although our interviewees discussed some of the drawbacks of telemedicine, they also offered solutions. As a solution to the provider-related issues, they suggested that the service be provided by a well-qualified and trained provider with excellent communication skills. Moreover, a HCP mentioned the importance of having an ethical background and using HIPAA-compliant platforms to secure patients’ privacy. The most important solution stated was using telemedicine to triage patients’ needs which may eventually lead to decreasing the workload on the healthcare providers. Furthermore, providing a stable internet connection and technical support for the providers and patients can overcome any technical difficulties. The HCPs also recommended forming an information technology (IT) team within the hospital or polyclinic. Regarding patient-related problems, it was suggested to schedule appointments for online visits, which could subsequently be entered into tables and assigned to each patient at a particular time. Moreover, healthcare professionals recommended sending instructions to patients on how to use the applications before conducting the visits. As one HCP stated, *“we sent them instructions on how to use the program and if any problem happens, how to solve the problem” (P22, allied health).*

#### Theme 6: benefits of telemedicine

In this theme, we identified the benefits of televisits to encourage its implementation. The HCPs interviewed mentioned the reduction in infection exposure as a clear benefit of televisits. In addition, the participants stated saving their time and decreasing their burden as a benefit which opposes what was mentioned by other HCPs that it increases their workload. Additionally, many professionals reported using televisits to triage patient needs and decide if in-person encounters are required, as one of them declared, *“the patients… great percentage of them do not need emergency so we will guide them instead of coming to the hospital and returning” (P19, physician).* The patients benefiting from televisits included follow-up cases that need remote monitoring and chronic-controlled patients, but not acute conditions. Furthermore, many HCPs reported that the patients who would benefit the most are those who are unable to drive, live in remote areas, or try to avoid adverse weather conditions.

## Discussion

This study uses mixed methods to explore HCPs’ perceptions of telemedicine use in practice in Kuwait. The mixed methods design was best suited to understand the experiences of healthcare professionals since the questionnaire included a variety of aspects that helped in generating themes after interviewing the HCPs. Overall, our findings show that telemedicine is viewed as a beneficial, easy, and efficient way of healthcare delivery that could be a suitable alternative to traditional methods of care delivery. Our study explored HCPs’ experiences of using televisits to provide and access healthcare services in Kuwait during and after the COVID-19 pandemic. The healthcare professionals found telemedicine beneficial in many aspects; however, this was accompanied by an acknowledgment of barriers and challenges. Time and cost-saving spurred telemedicine use and the baseline HCPs' high intention rate to use telemedicine paves the way for official incorporation into healthcare systems.

Findings from the present study show that knowledge of telemedicine, previous experience, and perceived usefulness or ease of use are the main driving forces of healthcare providers' telemedicine use intention. Participants in the current study had moderate to high attitudes towards telemedicine. This was consistent with a study, where participants cited self-care improvement, hospitalizations reduction, and provision of high-quality care using telemedicine [16]. The interviews showed that although some HCPs accepted telemedicine, others were resistant. In Ayres et al. (2021), resistant HCPs focused on the limitations of telemedicine and perceived it as a temporary substandard substitute to in-person visits [17]. Also, many interviewees mentioned reduction in risk of infection as a benefit that encouraged them to implement televisits in their practice [18]. Less than a quarter of our HCPs were concerned for privacy violation when using telemedicine, however, more participants in older studies cited this issue [8, 19]. Our interviews and other studies suggested using HIPAA-compatible platforms which are easy to use, help in reaching patients, and maintain privacy [9, 17, 18].

The current study showed that the majority of participants were knowledgeable about telemedicine, which was in line with a similar study conducted in Norway and Lithuania [16], and this knowledge may have enhanced participants’ telemedicine use intention. Previous experience with telemedicine appeared to be positively correlated with HCPs' use preference, where almost three-quarters of phase 1 participants who have a previous telemedicine experience preferred using it in the future as well. This correlation was consistent with Ruiz Morilla et al. (2017), who noted a positive view in those who utilized telemedicine in their workplace [20].

By assessing correlations, use intention was modulated by knowledge and experience with telemedicine, perceived usefulness, ease of use, and attitude. Attitude towards telemedicine was the most significant factor, where 82.8% of those with high attitude scores had a high use intention score as well. This was consistent with one study that showed a positive effect of attitude on use intention [13].

The interviews in the present study showed that good communication skills, patience, and sympathy are the components of a trustful relationship between HCPs and patients. In addition to the previous qualities, empathy and emotional support were also mentioned in other studies [21]. In our interviews, we only asked the participants who had already tried telemedicine before if they thought that training was required, and 8 out of 11 said yes. One healthcare professional noted that the MOH system is complicated and requires training before usage; however, those who opposed training emphasized the ease with which platforms may be used. From this finding we can infer the importance of providing education and training in this field to improve providers' opinions and provide a technologically capable working team, thus facilitating telemedicine implementation [20].

Results from interviews revealed that the lack of physical examination was the most mentioned barrier by HCPs as they could not read patients' body language, make eye contact, or complete an objective examination. These findings were similar to current literature that addressed the importance of physical examination and non-verbal communication [21, 22]. Patient-related factors such as their age, hearing, visual, as well as language abilities were found to be barriers to conducting successful televisits [23]. Furthermore, patients’ level of education is critical, as not all of them can use applications or gain access to this type of service. Unlike other studies that showed an effect of age [20] on perceived usefulness and gender on attitude [24], sociodemographic characteristics had no significant effect on the results of this study. Additionally, some patients refuse to appear on camera because they fear being recorded. However, a study conducted in Kuwait looked at patients’ perceptions of virtual healthcare services and found that 24.3% were concerned about privacy, while 48.5% believed video calls were secure [9].

In the present study, we assessed whether providers thought virtual visits dehumanize interpersonal interactions or make them uncomfortable. Less than a quarter perceived dehumanized patient care, while half of the respondents felt comfortable using telemedicine. This might be explained by the familiarity with technology generally as it is implemented in almost all daily activities and used by healthcare providers during conferences and continuous medical education programs.

Findings from this study show that many participants were uncertain whether telemedicine leads to increased cost by increasing workload or not. When workload was further investigated through interview, some perceived increased workload using telemedicine, while others claimed it saves time. Another study conducted in 2021 also cited increased workload [25]. On balance, other studies expected more cost and time saving because of reduced travel time and transportation expenses [26, 27].

Our study revealed several benefits of telemedicine. One promising finding is the HCPs perceived high telemedicine usefulness. Amodt et al. (2019) produced similar findings where participants cited the feasibility of timely feedback and decreased workload by enabling the assessment of more patients in less time. Enhanced communication was also cited by Crilly et al. (2019), which noticed improved pharmacist-patient relationships after implementing telemedicine in community pharmacies. Interviews revealed that HCPs use televisits to triage patients and meet patients’ needs, which is a desired benefit as well as a facilitator of telemedicine implementation.

Nearly half of HCPs highly believed telemedicine is easily used, which exceeds the results of other studies. In this study, 54.9% find it easy to control telemedicine technology and get it to do what they command, but in Mammen et al. (2018), 46.9% faced technical problems while applying this technology [14]. Another discussed aspect is the flexibility of telemedicine stated by 48.9% of HCPs, yet inflexibility was stated in Helou et al. (2020) by physicians who could not manage consultation times adequately. Another mentioned inflexibility was the inability to retrieve patients' records [7].

Further elaboration during interviews were done to assess how these barriers could be overcome and increase use intention. One point is the need for highly qualified HCPs who are aware of strategies promoting a strong relationship with patients to enhance patient acceptance of telemedicine. Another point addressed how essential it is to have an IT team available at healthcare institutions, to ensure continuity of care and solve technical issues [27, 28]. A previous study also conducted in Kuwait addressed the importance of cooperation between HCPs and IT team [29].

### Limitations

Using self-reported online measures adds selection bias to the results. However, the global COVID-19 outbreak and the mandatory public lockdown in Kuwait made this research methodology feasible. The lack of HCPs email lists or general population electoral roll impaired our ability to distribute questionnaires widely and this may hinder our ability to get adequate representation for all sectors of health professionals.However, the authors used multiple methods to reach out to healthcare providers, such as personal contact and using social media.

Regarding the qualitative phase of this study, although the study aimed to address the HCPs perceptions about the implementation of telemedicine in their practice, half of our interviewees had not used telemedicine before, which means that their perceptions may change when they use it. Furthermore, like most of the qualitative studies, our study’s sample size was small, but the themes derived from the data were representative due to the strong saturation of the data that emerged.

## Conclusion

The COVID-19 pandemic resulted in a dramatic shift of traditional in-person visits to virtual healthcare services. HCPs supported the incorporation of telemedicine in their practice in Kuwait, acknowledging the benefits and barriers of its implementation. Moreover, they suggested solutions to overcome the barriers. Future studies may consider comparing the perceptions of both patients and HCPs about telemedicine to evaluate their overall satisfaction and quality of care.

### Supplementary Information

**Additional file 1. **Phase 1 questionnaire.

**Additional file 2. **Phase 2 interview guide.

## Data Availability

The datasets analyzed during the current study are available from the corresponding author on reasonable request.

## Abbreviations

COVID-19: Coronavirus Disease 2019
ENT: Ear, Nose, Throat
HCP: Healthcare Provider
HIPAA: Health Insurance Portability and Accountability Act
IT: Information Technology
IQR: Interquartile Range
MOH: Ministry of Health
P: Participant
QSS: Qualtrics Survey Software
SPSS: Statistical Package for the Social Sciences
WHO: World Health Organization
