# Supplementary material for: Exploring healthcare providers’ perspectives on virtual care delivery: insights into telemedicine services
Source: BMC Health Serv Res. 2024 Jan 2;24:1. doi: 10.1186/s12913-023-10244-w (PMC10763041; doi:10.1186/s12913-023-10244-w)
Supplement: Supplementary file 1 — Additional file 1. Phase 1 questionnaire. [file 12913_2023_10244_MOESM1_ESM.docx]

**Exploring Healthcare Providers' Perspectives on Virtual Care Delivery: Insights into Telemedicine Services**

Research questionnaire for healthcare providers

1. **Section one:**
2. What is your age? (years)
3. Gender:
   1. Male
   2. Female
4. Nationality:
   1. Kuwaiti
   2. Non-Kuwaiti
5. How would you rate your communication skills in Arabic?
   1. Very good
   2. Good
   3. Neither good nor poor
   4. Poor
   5. Very poor
6. How would you rate your communication skills in English?
   1. Very good
   2. Good
   3. Neither good nor poor
   4. Poor
   5. Very poor
7. What is your profession?
   1. Physician
   2. Pharmacist
   3. Nurse
   4. Other, specify
8. Working location:
   1. Governmental hospital
   2. Private hospital
   3. Polyclinic
   4. Other, please specify:
9. Working health area?
   1. Hawally
   2. Al-Asimah (Capital)
   3. Ahmadi
   4. Farwaniya
   5. Jahra
   6. Mubarak Al-Kabeer
   7. Al-Sabah
10. Years of experience?
    1. Dropdown menu from <1 year to ≥ 40 years
11. What is your job title?
12. Which working shift are you working? (you can choose more than one)
    1. Day shift
    2. Night shift
    3. Afternoon
13. Highest degree obtained:
    1. Diploma
    2. Bachelor's degree
    3. Master's degree
    4. Ph.D.
    5. Other, please specify
14. **Section two:**
15. Telemedicine is the practice of medicine using technology to deliver care at a distance. Based on that definition please tell me which of the following statements best describes you?
    1. I have never heard of telemedicine technology
    2. I have heard of telemedicine, but don’t know much about it.
    3. I know about telemedicine technology but have not used it yet.
    4. I have used telemedicine before.
16. At this time:
    1. I prefer not to use telemedicine.
    2. I prefer to use telemedicine.
    3. I am not sure and need more information.
17. **Section three:**
18. The following statements refer to whether telemedicine can enhance patient care. Please read each statement carefully, and then choose only one answer for each statement.

| Statements: | Strongly disagree | Disagree | Uncertain | Agree | Strongly agree |
| --- | --- | --- | --- | --- | --- |
| 1. Using telemedicine would enable me to accomplish tasks more quickly. | 1 | 2 | 3 | 4 | 5 |
| 1. Using telemedicine would improve my job performance | 1 | 2 | 3 | 4 | 5 |
| 1. Using telemedicine in my job would increase my productivity. | 1 | 2 | 3 | 4 | 5 |
| 1. Using telemedicine would enhance my effectiveness on the job | 1 | 2 | 3 | 4 | 5 |
| 1. Using telemedicine would make it easier to do my job. | 1 | 2 | 3 | 4 | 5 |
| 1. I would find telemedicine useful in my job. | 1 | 2 | 3 | 4 | 5 |
| 1. Using telemedicine would improve communication on my job. | 1 | 2 | 3 | 4 | 5 |

1. **Section four:**
2. The following statements refer to whether a telemedicine system is easy to use. Please read each statement carefully, and then circle only one answer for each statement.

| Statements: | Strongly disagree | Disagree | Uncertain | Agree | Strongly agree |
| --- | --- | --- | --- | --- | --- |
| 1. Learning to operate telemedicine technology would be easy for me. | 1 | 2 | 3 | 4 | 5 |
| 1. I would find it easy to get telemedicine technology to do what I want it to do. | 1 | 2 | 3 | 4 | 5 |
| 1. My interaction with telemedicine technology would be clear and understandable. | 1 | 2 | 3 | 4 | 5 |
| 1. I would find telemedicine technology to be flexible to interact with | 1 | 2 | 3 | 4 | 5 |
| 1. It would be easy for me to become skillful at using telemedicine technology. | 1 | 2 | 3 | 4 | 5 |
| 1. I would find telemedicine technology easy to use. | 1 | 2 | 3 | 4 | 5 |

1. **Section five:**
2. Attitude toward telemedicine. Please read each statement carefully, and then circle only one answer for each statement.

| Statement: | Strongly disagree | Disagree | uncertain | agree | Strongly agree |
| --- | --- | --- | --- | --- | --- |
| 1. The use of telemedicine improves patient care by giving the provider more time with the patients. | 1 | 2 | 3 | 4 | 5 |
| 1. Telemedicine can be adapted to assist providers in many aspects of patient care. | 1 | 2 | 3 | 4 | 5 |
| 1. A telemedicine system offers providers a remarkable opportunity to improve patient care. | 1 | 2 | 3 | 4 | 5 |
| 1. Telemedicine technology represents a violation of patient privacy | 1 | 2 | 3 | 4 | 5 |
| 1. Telemedicine technology causes providers to give less time to quality patient care | 1 | 2 | 3 | 4 | 5 |
| 1. Telemedicine increases cost by increasing the provider’s workload. | 1 | 2 | 3 | 4 | 5 |
| 1. It takes as much effort to maintain patient records using telemedicine technology as it does by hand | 1 | 2 | 3 | 4 | 5 |
| 1. Telemedicine creates more problems than they solve in providing health care | 1 | 2 | 3 | 4 | 5 |
| 1. The use of telemedicine dehumanizes patient care | 1 | 2 | 3 | 4 | 5 |
| 1. Part of the increase in costs of health care is because of telemedicine | 1 | 2 | 3 | 4 | 5 |
| 1. Confidentiality will not be sacrificed by using telemedicine. | 1 | 2 | 3 | 4 | 5 |
| 1. I would be comfortable using telemedicine. | 1 | 2 | 3 | 4 | 5 |
| 1. Working with telemedicine technology would make me very nervous. | 1 | 2 | 3 | 4 | 5 |
| 1. I feel threatened when others talk about telemedicine. | 1 | 2 | 3 | 4 | 5 |
| 1. Telemedicine technology does not scare me at all. | 1 | 2 | 3 | 4 | 5 |
| 1. I feel hostile toward using telemedicine. | 1 | 2 | 3 | 4 | 5 |
| 1. Telemedicine makes me feel uneasy and confused | 1 | 2 | 3 | 4 | 5 |
| 1. I have a lot of self-confidence when it comes to working with telemedicine | 1 | 2 | 3 | 4 | 5 |
| 1. Confidentiality is nearly impossible if patient records are used during telemedicine. | 1 | 2 | 3 | 4 | 5 |
| 1. Providing health care does not lead itself to use telemedicine | 1 | 2 | 3 | 4 | 5 |
| 1. Telemedicine would make providers’ jobs easier. | 1 | 2 | 3 | 4 | 5 |

1. **Section six:**
2. Intention to use telemedicine to provide patient care. Please read each statement carefully, and then circle only one answer for each statement.

| Statements: | Strongly disagree | Disagree | Uncertain | Agree | Strongly agree |
| --- | --- | --- | --- | --- | --- |
| 1. I intend to use telemedicine with my patient care and management when it is available in my clinic or hospital. | 1 | 2 | 3 | 4 | 5 |
| 1. I intend to use telemedicine technology to provide healthcare services to patients as often as needed. | 1 | 2 | 3 | 4 | 5 |
| 1. I intend NOT to use telemedicine in my patient care and management routinely | 1 | 2 | 3 | 4 | 5 |
| 1. Whenever possible, I intend NOT to use telemedicine in patient care and management. | 1 | 2 | 3 | 4 | 5 |
| 1. To the extent possible, I would use telemedicine technology to do different things, clinical or non-clinical. | 1 | 2 | 3 | 4 | 5 |
| 1. To the extent possible, I would use telemedicine in my patient care and management frequently. | 1 | 2 | 3 | 4 | 5 |

1. You may provide your phone number if you are happy to be invited for an interview which will take about 10 minutes over the phone or a video call. The interview will allow us to learn more about the barriers to adopt this technology.
