# Supplementary material for: Exploring healthcare providers’ perspectives on virtual care delivery: insights into telemedicine services
Source: BMC Health Serv Res. 2024 Jan 2;24:1. doi: 10.1186/s12913-023-10244-w (PMC10763041; doi:10.1186/s12913-023-10244-w)
Supplement: Supplementary file 2 — Additional file 2. Phase 2 interview guide. [file 12913_2023_10244_MOESM2_ESM.docx]

**Interview questions guide and prompts**

Hello, Ph. …………….is speaking.

I am calling from Kuwait university faculty of pharmacy, to gather information about HCPs’ perception of healthcare services provided virtually (telemedicine).

You have volunteered by providing your phone number in the previous questionnaire last year.

- Would you mind answering a few questions at this time?
  - No → when can I call you back?

Month/day

Time?

- - Yes →

Thank you for agreeing to give your time for this interview as a follow up to your completion of the survey “Exploring Healthcare Providers' Perspectives on Virtual Care Delivery: Insights into Telemedicine Services”

This interview should take no longer than X minutes

This interview is recorded, are you okay with that?

If they said no: I'll stop recording now and start taking notes, but this will take extra time. Are you okay with that?

1. Where do you work and what is your job title? (Specialty, department, hospital)
2. Have you used e-health before? (if no, go to barriers except questions 7,8,9).
3. Tell me about a situation where you have tried to use e-health in your practice.
   1. What platform did you use?

(Zoom, teams, WhatsApp, phone call)

- 1. Do you usually use text messages, phone calls, or video calls?
  2. When do you use it?

(Only during the pandemic, during quarantine, stopped after quarantine, depends on the patient’s preference)

- 1. How often do you use it?

(Daily, 1-2 days/week, occasionally)

- 1. Are you willing to use video calls in future?

(If answer to b was either texts or phone calls)

- 1. What do you rate your overall experience with eHealth?

1. What motivated you as a healthcare provider to use e-health?

(Incentive, training, availability of an easy-to-use app)

1. In your opinion what are the requirements to have a successful incorporation of telemedicine into our healthcare system?

(Administrative support, good training, good patient-provider relationship)

**Barriers**

1. What is limiting you from using e-Health in your practice?

(I didn’t use it before, not available in practice)

1. What difficulties or challenges have you experienced when using e-Health?

(Cost, Communication, misunderstanding /misinterpreting information, time, technology…etc.)

1. How did you solve it?
2. Did you experience changes in the contact/bond with the patient?
3. Do HCPs require education before use and how did that happen in the past?

**Benefits**

1. From your point of view what are the benefits of implementing telehealth and who would benefit the most from it? and why?

(Reduced infection exposure, time-efficient for the provider, more frequent follow up visits)

1. From your experience with eHealth, do you find it same, better, or worse than in person visits?

(If the answer was no: Do you think eHealth is the same, better, worse ….)

1. What other e-services do you think pharmacists/physicians/nurses can potentially make a significant contribution to? (If is the answer was no: what e-services do you think can make a significant contribution?)
2. Do you know someone that uses eHealth? And can you give us his contact number?
3. Would you have any further suggestions or comments regarding this topic that have not been covered in this interview? If so, what are they please?
